# Population Genomic Insights Into Recent Nutria (*Myocastor coypus*) Invasion Dynamics

**DOI:** 10.1111/eva.70168

**Published:** 2025-11-24

**Authors:** Kristen D. Ahrens, Joshua M. Hallas, Antionette J. Piaggio, Kelly L. Carrothers, Valerie K. Cook, Michael R. Buchalski

**Affiliations:** ^1^ Wildlife Genetics Research Unit, Wildlife Health Laboratory California Department of Fish and Wildlife Sacramento California USA; ^2^ Mammalian Ecology and Conservation Unit, Veterinary Genetics Laboratory, School of Veterinary Medicine University of California Davis California USA; ^3^ Wildlife Genetics Laboratory, National Wildlife Research Center, Wildlife Services, Animal and Plant Health Inspection Service United States Department of Agriculture Fort Collins Colorado USA; ^4^ Genetics Research Laboratory California Department of Fish and Wildlife Sacramento California USA; ^5^ Beaver Restoration and Nutria Eradication Program California Department of Fish and Wildlife West Sacramento California USA

**Keywords:** biological invasion, mitochondrial DNA, *Myocastor coypus*, nutria, RADseq, single nucleotide polymorphism

## Abstract

Nutria ( *Myocastor coypus* ) are semi‐aquatic rodents native to South America, introduced to the USA for fur farming during the early twentieth century. This species' herbivory can cause extensive damage to agriculture and wetland ecosystems. Though declared eradicated from California, USA, in the 1970s, nutria populations were recently discovered in the state's Central Valley and subsequently the Sacramento–San Joaquin Delta, areas of agricultural and conservation significance. We report the use of a combination of nuclear single nucleotide polymorphisms (SNPs) and mitochondrial (mtDNA; cytochrome b locus) markers to characterize the source and demographic history of the current invasion, with the goal of informing eradication efforts. Our study is the first to develop a SNP dataset for nutria, utilizing 6809 loci to characterize genetic diversity in comparison to several potential source populations. Multivariate analysis and Bayesian clustering of the SNP dataset found the greatest similarity to invasive nutria in central Oregon, USA, with minimal genetic differentiation in the Central Valley excluding the leading edges of the invasion. Cytochrome b sequencing resulted in a single contemporary California haplotype shared with nutria in Oregon and Washington but also detected in museum samples from California fur farms predating eradication. Mantel tests found genetic differentiation among nutria in the Central Valley was best explained by ecological distance along rivers, while estimated effective migration surface (eems) analysis indicated gene flow was characterized by infrequent dispersal followed by rapid expansion in large, protected areas of emergent wetland habitat. These combined findings suggest contemporary California nutria represent a recent introduction that underwent rapid expansion. Our data further support treating the Central Valley as a single eradication unit while investing additional resources in targeting dispersal corridors to best achieve management goals. This study presents the first characterization of a regional nutria invasion within the larger context of global population and phylogenetics.

## 1 Introduction

The proliferation of invasive species resulting from human activities is considered one of the largest drivers of ecosystem degradation and loss of biodiversity globally (Mack et al. 2000; Blackburn et al. 2004; Sax et al. 2007; Gallardo et al. 2016). The introduction of invasive species can cause severe environmental degradation, resulting in a variety of economic losses to agriculture, forestry, and fisheries resources (Pimentel et al. 2000; Castilla et al. 2005; Shackleton et al. 2019; Vaissière et al. 2022). Among invasive species, non‐native mammals are considered one of the greatest threats to native species and environmental quality (Courchamp et al. 2003; Blackburn et al. 2004; Dueñas et al. 2021). Eradication has therefore become an important tool for ecosystem restoration and the protection of agricultural systems (Bomford and O'Brien 1995; Carter and Leonard 2002; Baker 2010; Jones et al. 2016). However, these efforts can fail due to a lack of information on the origin, demography, and movement patterns of the target species (Abdelkrim et al. 2007; Holmes et al. 2015; Kappes et al. 2019). Genetic analysis is a useful strategy for deciphering the dynamics of an invasion and prioritizing management actions such as surveillance and eradication (Fraser et al. 2013; Cristescu 2015; Browett et al. 2020; Matheson and McGaughran 2022; McGaughran et al. 2024).

There are several ways analyses of genetic diversity within invasive populations can inform management and control (Lee 2002; Hampton et al. 2004; Le Roux and Wieczorek 2009; Lawson Handley et al. 2011). Genetic data can elucidate potential source populations for recent invasions, which is important for identifying and disrupting active immigration routes (Ficetola et al. 2008; Estoup and Guillemaud 2010; Cristescu 2015). Genetic data can also help distinguish whether individuals represent a recolonization event or are survivors of a previous eradication effort (Abdelkrim et al. 2007). Insights can be obtained into demographic history using metrics of genetic diversity, such as heterozygosity, relatedness, and extent of inbreeding (Drygala et al. 2016; Zalewski et al. 2016; Titus et al. 2022). Characterization of population genetic structure and gene flow allows for evaluation of connectivity within an invaded area, as genetic discontinuities often reflect barriers to dispersal (Estoup and Guillemaud 2010). Managers can use such information to identify ‘eradication units,’ that is interconnected habitat patches that must be eradicated at the same time to avoid subsequent recolonization (Robertson and Gemmell 2004; Abdelkrim et al. 2005; Adams et al. 2014). Eradication units can then be prioritized based on criteria such as recolonization risk, allowing resources to be allocated more effectively (Abdelkrim et al. 2005). However, the promise of genetic data to provide management insights can be complicated by the fact that many invasive species may have low genetic diversity due to founder effects (Dlugosch and Parker 2008; Dlugosch et al. 2015; Estoup et al. 2016; Buchholz et al. 2023). Therefore, genotyping methods that employ thousands of loci are highly beneficial for obtaining sufficient data resolution. Restriction site‐associated DNA sequencing (RADseq) methodologies are particularly powerful for generating sequences aiding in the discovery of large numbers of single nucleotide polymorphism (SNP) loci in understudied species with limited genomic resources (i.e., no reference genome; Andrews et al. 2016; Browett et al. 2020). Additionally, high‐density SNP datasets allow for high‐resolution measures of population structure, modeling of landscape genetic connectivity, and other metrics of genetic diversity.

**FIGURE 1:**
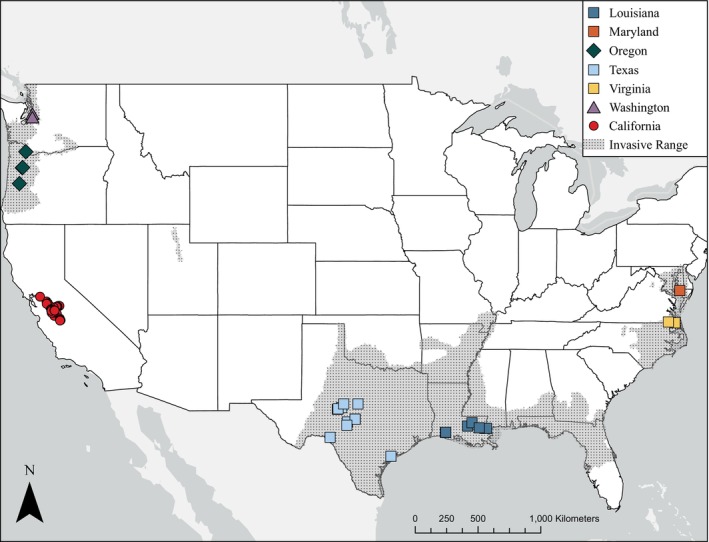
Locations for samples used in this study superimposed on approximate nutria invasive range in the USA (U.S. Geological Survey 2018; data based on 2001 conditions). Note that nutria populations in Chesapeake Bay, Maryland, have been deemed successfully eradicated since 2015.

Native to South America, nutria or coypu ( *Myocastor coypus* ; Molina 1782) are large, semi‐aquatic rodents in the family Echimyidae (Woods et al. 1992). Once valued for fur production and later intentionally introduced for weed control, nutria were widely exported to every continent except Antarctica and Australia during the early 20th century (Carter and Leonard 2002). In most areas, fur‐farmed nutria escaped or were intentionally released, resulting in numerous invasive populations across the globe (Carter and Leonard 2002; Bertolino and Genovesi 2007; Hong et al. 2015). In the USA, nutria are considered a pest species in 19 states (U.S. Geological Survey 2018; Figure 1) as their voracious herbivory can destroy marsh vegetation and agricultural crops, and their burrows undermine levees and other water control structures (Evers et al. 1998; Carter et al. 1999; Carter and Leonard 2002; Johnson Randall and Foote 2005). These behaviors have been attributed to numerous forms of ecosystem damage, including reduction in native plant biomass and diversity (Taylor and Grace 1995; Evers et al. 1998; Baroch et al. 2002; Prigioni et al. 2005), disruption to wetland hydrology (Ford and Grace 2002), and increased erosion and destruction of riverbanks (Carter et al. 1999; Panzacchi et al. 2007). Therefore, the International Union for Conservation of Nature (IUCN) considers nutria one of the worst invasive species globally (Lowe et al. 2000; Kopf et al. 2017). Further, nutria can carry a variety of zoonotic pathogens that place both domestic livestock and wildlife at risk (Howerth et al. 1994; Park et al. 2014; Fratini et al. 2015). Global impacts from nutria invasions have prompted many governments to institute nutria‐control programs (Gosling and Baker 1989; Panzacchi et al. 2007; Pepper et al. 2018). However, eradication efforts prove challenging as nutria can occupy a wide variety of wetland habitats, have a broad diet, and a high reproductive rate (Woods et al. 1992; Bertolino et al. 2005).

**FIGURE 2:**
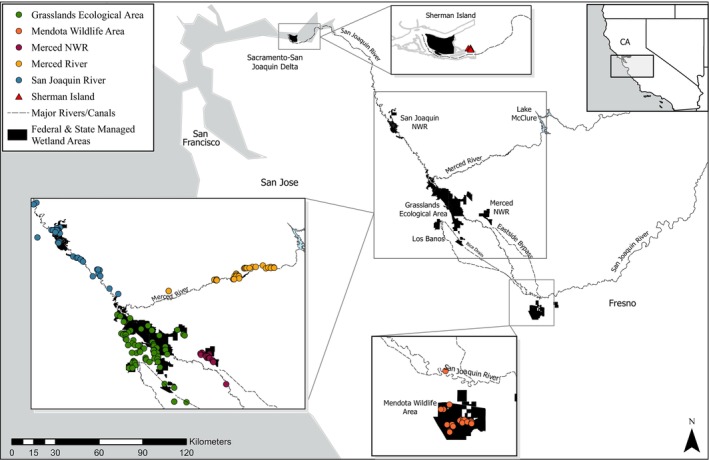
Map of managed wetland areas, major drainages, and nutria sampling locations within the Central Valley and Sacramento–San Joaquin Delta in California, USA, color coded by management unit (MU).

The first nutria imported to the United States were to establish a fur farm at Elizabeth Lake, California, in 1899 (Evans 1970). Though this initial farm was unsuccessful, subsequent importations established multiple farms over the next several decades, and by 1940 California had a small feral population (Schitoskey Jr. et al. 1972). The eventual collapse of the nutria fur trade and recognition of the dangers nutria posed as a pest species to agriculture and natural ecosystems resulted in coordinated eradication efforts in California, and by the 1970s, the feral population was reported to be eradicated (Deems and Pursley 1978). However, in 2017, approximately 20 nutria were depredated from the Grasslands Ecological Area in California's Central Valley (Figure 2), followed by rapid demographic and range expansion during 2018–2020. The California Department of Fish and Wildlife (CDFW) initiated an emergency response in 2018, forming the Nutria Eradication Program to live‐trap, dispatch, necropsy, and genetically sample nutria, with > 3000 animals culled at the time of this study in 2022. Given the absence of nutria detections between the 1970s and 2017, and the rapid expansion following discovery, contemporary California nutria are hypothesized to have descended from a small introduction of animals of unknown origin. This is in contrast to the less likely scenario that a remnant population has persisted undetected since the 1970s in intensively managed wetland areas.

The Central Valley and Sacramento‐San Joaquin Delta comprised the current invasive range in California at the time of sampling (Figure 2), with nutria rapidly colonizing the little remaining protected emergent marsh and riparian wetlands. Over 90% of these habitats have been lost in the last 200 years through land reclamation and are vital to the long‐term persistence of waterfowl and other wetland‐dependent species (Gilmer et al. 1982; Shuford et al. 1998; Fleskes et al. 2018). The Central Valley is also the largest area of agriculture in California and one of the most productive agricultural regions in the world, producing over 25% of food in the USA in an area roughly 52,000 km^2^ which represents a $20 billion industry and accounts for ~13% of the total USA agricultural exports (Great Valley Center 2005; California Department of Food and Agriculture 2024). The surface hydrology of this landscape has been extensively altered to maximize water reclamation for agriculture, resulting in numerous canals that may also serve as nutria habitat or facilitate dispersal, as well as flood control levees that may be damaged by nutria (Kuhn and Peloquin 1974). Further, the Mediterranean climate of the region provides little seasonal control of nutria population growth as mild winters facilitate year‐round breeding, while climate shifts in California between record high precipitation and megadrought further complicate management strategies as it pertains to habitat abundance and connectivity (i.e., wetland inundation; Baroch et al. 2002; Denena et al. 2003; Hong et al. 2015). All of the above factors could result in the Central Valley becoming one of the most severely impacted areas of nutria invasion in North America. Thus, there is an urgent need to understand nutria invasion dynamics, as well as to develop and implement effective management strategies for eradication.

Genetic analyses at various spatial scales are necessary for developing nutria eradication strategies and identifying the source of new invasions (Lee 2002; Le Roux and Wieczorek 2009). For example, despite consensus regarding the global threat nutria pose to agriculture and native wetlands, there has been no research characterizing the distribution of genetic variation on a continental or global scale. In California, there are no records of the number or origin of imported animals. The absence of such information makes it difficult to differentiate reinvasion from expansion of a remnant population. Further, for many invasive species, eradication programs fail due to reinvasion from neighboring areas (Abdelkrim et al. 2007). Eradication of nutria requires the isolation of invaded areas from sources of new immigrants (Carter and Leonard 2002), making it necessary to characterize landscape‐scale patterns of dispersal. Genetic methods can provide such inferences without the need for time‐consuming and costly traditional wildlife survey methods such as mark‐recapture or radiotelemetry. To adequately address the scope of the invasion, there is a need for genetic data regarding the source of contemporary California nutria, as well as genetic diversity and structure among populations for the purpose of inferring demographic history and landscape connectivity (Lee 2002; Le Roux and Wieczorek 2009; Fitzpatrick et al. 2011) in order to implement effective eradication strategies.

Here, we assessed genetic variation in nutria in California and report the first use of SNP markers to characterize this pest species that has challenged the control efforts of managers worldwide for decades. Our approach combined RADseq SNP discovery and mtDNA sequencing with systematic sampling of the invaded area. We also included museum samples from California fur‐farmed animals predating nutria eradication in the 1970s, as well as samples from other invasive populations in the USA. With this study design, we had the following objectives: (1) identify the source of the contemporary California nutria invasion, (2) characterize genetic diversity to infer the demographic history of the recent invasion, (3) elucidate population structure and landscape patterns of gene flow to better understand dispersal throughout the invaded area, (4) facilitate genetically informed eradication strategies, and (5) create both national and global comparative genetic datasets to aid in the control of nutria. The recent re‐emergence in California is an ideal model for understanding early nutria invasion dynamics. This study provides a genetic foundation for the development of management strategies for California, but also global nutria invasions more broadly. This represents the first genomic study of invasive nutria and the broadest geographic scale across which nutria have been genetically characterized.

## 2 Materials and Methods

### 2.1 Study Area and Sample Collection

We divided the California invasion into discrete management units (MUs), as determined by the CDFW Nutria Eradication Program. MUs were largely defined by the spatial distribution of suitable habitat and separation between occupied habitat patches. This included six distinct MUs, which were used to subset individuals for downstream analyses: (1) the Mendota Wildlife Area (WA) at the southern edge of the invasion; (2) the Grasslands Ecological Area, a complex of managed wetlands encompassing the San Luis National Wildlife Refuge (NWR), Great Valley Grasslands State Park, Los Banos and North Grasslands WAs, and adjacent wetlands; (3) Merced NWR, which, while part of the Grasslands Ecological Area, was analyzed separately given its geographic separation and distinct management considerations; (4) the Merced River downstream of Lake McClure; (5) the San Joaquin River between the Grasslands Ecological Area downstream to and including San Joaquin NWR; and (6) Sherman Island in the Sacramento‐San Joaquin Delta, representing the northern edge of the invasion and recent range expansion at the time of sampling (Figure 2; Table 1; Table S1). MUs 2–5 represent the core invaded area. MUs are often connected by either mainstem river corridors (Merced and San Joaquin rivers) or canals. Contemporary samples from California (*n* = 267) were acquired through nutria eradication efforts conducted by CDFW throughout the study area. Field crews live‐trapped and culled nutria from 2020 to 2022 under the Department's authority as the trustee for wildlife management in the state, CA Fish & Game Code § 1802 (2015). Carcasses were transported to field stations for necropsy and genetic sampling of tail tips or organ tissue.

**TABLE 1:** Sample sizes (*n*) and summary statistics for contemporary California nutria. Expected heterozygosity (*H*_E_), inbreeding coefficient (*F*_IS_), and mean KING‐robust kinship estimates are presented by management unit (MU). Pairwise genetic differentiation between MUs was calculated as conditional genetic distance (*cGD*) in the upper triangle and *F*_ST_ in the lower triangle.

Management unit	*n*	*H*_E_	Mean relatedness	*F*_IS_	Mendota WA	Grasslands Ecol. Area	Merced NWR	Merced River	San Joaquin River	Sherman Island
					*cGD* / Weir & Cockerham *F*_ST_					
Mendota WA	43	0.28	0.02	−0.049	—	2.78	0.84	1.93	3.80	3.52
Grasslands Ecol. Area	91	0.33	−0.031	−0.0064	0.08	—	3.61	0.85	1.02	3.59
Merced NWR	35	0.31	−0.051	0.0011	0.06	0.03	—	2.77	4.64	4.36
Merced River	45	0.33	−0.055	0.016	0.10	0.03	0.06	—	1.87	3.70
San Joaquin River	46	0.32	−0.086	0.04	0.10	0.03	0.05	0.05	—	2.57
Sherman Island	7	0.20	0.15	−0.20	0.26	0.17	0.20	0.20	0.16	—
Mean		0.30	−0.053	−0.033						

To place the genetics of the contemporary California invasion in context with other invasive populations throughout the United States, we obtained nutria tissue (*n* = 40; Figure 1) from federal and state wildlife management agencies and museum collections (Table S1), including Louisiana (*n* = 11), Texas (*n* = 13), Maryland (*n* = 3), Virginia (*n* = 2), Oregon (*n* = 8), and Washington (*n* = 3). Lastly, we sampled museum skulls and pelts from nutria predating the 1970s California eradication (*n* = 11; Table 2), which we refer to as historical samples.

**TABLE 2:** Historical California samples sequenced at mitochondrial DNA loci. Locations denoted as ‘fur farm stock’ lacked exact location data but originated within California.

Institution	Sample ID	Sample type	Collection date	Location (CA)	Cyt *b*/D‐loop haplotype
Museum of Endothermic Vertebrates, California State University, Sacramento	CSUS‐142	Paw pad, tooth	1953	Oakdale	B/4
Museum of Vertebrate Zoology, University of California, Berkeley	MVZ:Mamm:118893	Pelt, tooth	1953	Hayward	A/1
Natural History Museum of Los Angeles County	LACM:Mamm:004824	Bone	1936	Covina	D/16
Museum of Wildlife and Fish Biology, University of California, Davis	WFB‐2491z	Paw pad, turbinate	1954	Fur farm stock	A/1
Museum of Wildlife and Fish Biology, University of California, Davis	WFB‐1625z	Paw pad, turbinate	1951	Stanislaus Co.	B/4
Butte County Dept. of Weights and Measures	BC‐Sam	Paw pad	Unknown	Butte Co.	D/‐
Aryan Roest Mammal Collection, Biological Sciences Dept., California Polytechnic State University, San Luis Obispo	SLO‐M‐561	Turbinate	1956	San Luis Obispo Co.	C/3
California Academy of Sciences	CAS:MAM:26583	Turbinate	1960	Fur farm stock	‐/3
California Academy of Sciences	CAS:MAM:26584	Turbinate	1960	Fur farm stock	C/‐
California Academy of Sciences	CAS:MAM:32250	Turbinate	1960	Fur farm stock	C/3
California Academy of Sciences	CAS:MAM:32251	Turbinate	1960	Fur farm stock	C/3

*Note:* Haplotyping naming convention followed previously published NCBI GenBank sequences for Cyt *b* (Kawamura et al. 2018) and D‐loop (Ibañez et al. 2021). Novel haplotypes C and D are available on GenBank (accession numbers PX240733 and PX240734).

### 2.2 Laboratory Methods

#### 2.2.1 DNA Extraction

For contemporary samples, we extracted either tail or organ tissue using the Mag‐Bind Blood and Tissue DNA HDQ kit following manufacturer guidelines (Omega Bio‐tek Inc., Norcross, GA, USA) performed on a Hamilton Microlab NIMBUS96 automated liquid handler (Hamilton Company, Reno, NV, USA). Negative controls were included for each set of extractions to detect contamination. We quantified the concentration of genomic DNA in duplicate using a SpectraMax M3 Absorbance Reader (Molecular Devices, San Jose, CA, USA) and AccuBlue Broad Range dsDNA Quantitation Kit (Biotium Inc., Fremont, CA, USA). To ensure DNA quality, we ran a 1% agarose gel stained with Invitrogen SYBR Safe at 80 V for 70 min.

We processed historical samples in an AirClean 600 Workstation (AirClean Systems, Creedmoor, NC, USA) operated in a laboratory space dedicated to trace DNA methods. We implemented protocols for preventing cross‐contamination, as outlined in Carrothers et al. (2021), including workstation decontamination steps between samples of 20% bleach treatment and 30‐min UV light exposure. We extracted pelt samples using the Omega Mag‐Bind Blood & Tissue DNA HDQ kit (Omega Bio‐tek Inc., Norcross, GA, USA) following the manufacturer protocol for tissues, with the addition of 10 μL of 1 M DTT per sample. For skulls, we selected either a tooth or turbinate, decontaminated the outer surface with 20% bleach, followed by 70% EtOH, sanded the surface using a handheld Dremel tool, and discarded the surface material before sample collection. We then sanded the decontaminated surface until approximately 75 mg of fine powder was collected in duplicate. We followed extraction methods described by Carrothers et al. (2021), which included a 24‐h demineralization step with 0.5 M EDTA pH 8.0, followed by extraction on an EZ1 Advanced XL extraction robot (QIAGEN Inc., Valencia, CA, USA) according to the manufacturer protocol for large‐volume samples. We then combined extracts from each duplicate, concentrated the DNA using a Vacufuge Plus (Eppendorf SE, Enfield, CT, USA) at 30°C, and reconstituted the purified DNA in 70 μL of water.

#### 2.2.2 Mitochondrial DNA Sequencing

To maximize our success sequencing the DNA of degraded museum samples, we designed nutria‐specific primers for two short segments of the mitochondrial DNA (mtDNA) cytochrome b (Cyt *b*) gene. We used Primer3, the National Center for Biotechnology Information (NCBI) Primer‐BLAST tool (Rozen and Skaletsky 1999; Ye et al. 2012), and whole mitogenome sequences acquired from NCBI GenBank (Accession numbers NC 035866.1, MH 182628.1, MF 325938.1, KU 892780.1) to design primers that amplify two contiguous fragments with overlapping primer‐binding sites, 358 bp and 293 bp in length. Primer sequences and amplicon lengths are described in Table 3. All primers were purchased from Integrated DNA Technologies (IDT; Coralville, Iowa, USA).

**TABLE 3:** Nutria‐specific primers designed for amplification of two contiguous cytochrome *b* mitochondrial DNA loci. Primer binding sites for CytB1‐14981‐R and CytB2‐14962‐F overlap by 19 bp. Displacement loop (D‐loop) primers were provided in Ibañez et al. (2021).

Oligo	Locus	Product Size (bp)	Sequence (5′–3′)
CytB1‐14624‐F	Cyt *b*	358	AGCTATCCCTTACATCGGTTCT
CytB1‐14981‐R			TGGTTTGATGTGGGGAGGTGTA
CytB2‐14962‐F	Cyt *b*	293	CACCTCCCCACATCAAACC
CytB2‐15254‐R			AATGCAAAAGTAGGAAACAGATGCT
Mco‐F	D‐loop	528	GAAACGGAGCACTACCCTCC
Mco‐R			CGAGATGTCTTATTTAAGGGGAAC

We combined Cytiva Illustra Hot Start Mix RTG formula (Uppsala, Sweden) with 2 μL of DNA template, 21 μL of H_2_O, and 1 μL of each forward and reverse primer (10 μM) for each polymerase chain reaction (PCR) for a 25 μL reaction. For historical samples, we followed the same protocol but increased the volume of template to 4 μL and decreased the H_2_O volume to 19 μL. Thermocycling conditions for Cyt *b* amplification were as follows: an initial step of 95°C for 5 min; followed by 36 cycles of 95°C for 30 s, 58°C for 45 s, and 72°C for 30 s; with a final extension step of 72°C for 5 min. We also amplified a 514 bp fragment of the mtDNA displacement loop (D‐loop) locus using primers and the thermocycling conditions provided in Ibañez et al. (2021). A negative control was included for each thermocycler run.

We purified PCR products using Applied Biosystems Inc. (ABI) ExoSAP‐IT and sequenced using the ABI BigDye Terminator v3.1 Cycle Sequencing Kit following manufacturer protocols (Foster City, CA, USA). We performed Sanger sequencing on an ABI 3500xL Genetic Analyzer with a 50 cm capillary array, POP‐7 polymer, and default instrument settings. We sequenced each sample in both forward and reverse directions to generate a consensus sequence. Each historical sample was sequenced in duplicate, resulting in a consensus sequence generated from two forward and two reverse sequences.

#### 2.2.3 Illumina Library Preparation and Sequencing

We implemented genotyping‐by‐sequencing (GBS) protocols modified from Elshire et al. (2011) to simultaneously discover and genotype thousands of SNP loci in our contemporary samples. We digested 300 ng of normalized genomic DNA (30 ng/μL) from each sample in a 20 μL reaction with 0.05 μL of NEB High Fidelity PstI restriction enzyme (200 U/μL) for 2 h at 37°C (New England Biolabs, Ipswich, Massachusetts, USA). Adapters and inline, single‐indexed barcodes (IDT; Coralville, Iowa, USA) were ligated to the digested DNA, and PCR amplification was performed across four replicate wells for each pooled library and then combined. Each PCR replicate consisted of a 50 μL reaction of 10 μL of pooled and purified library, 25 μL of NEB Taq 2X Master Mix, 1 μL of each forward and reverse primer (25 μM), and 13 μL of water. We used the QIAGEN QIAquick PCR Purification Kit (Valencia, CA, USA) to remove adapter and primer dimers following both ligation and PCR steps. We pooled 96 samples per lane across three lanes, with an initial lane of 48 pooled samples to test for adequate depth of coverage. As with genomic DNA, we quantified libraries using the Spectramax M3 and AccuBlue dsDNA Quantitation Kit. The pooled libraries were run on a Bioanalyzer (Agilent Technologies, Santa Clara, CA, USA) to confirm the absence of dimers and verify the appropriate library fragment size. We sent libraries to the UC Davis Genome Center, DNA Technologies and Expression Analysis Core for paired‐end 150 bp short‐read sequencing on the Illumina HiSeq4000 platform. We tested for lane effects by re‐sequencing 2–3 replicate individuals on each Illumina sequencing lane.

### 2.3 De Novo Assembly, Variant Calling and Filtering

We used gbsx v.1.3 (Herten et al. 2015) for demultiplexing and adapter trimming. Because a reference genome is not available for nutria, we created a de novo assembly following the dDocent pipeline (Puritz, Hollenbeck, and Gold 2014; Puritz, Matz, et al. 2014) which employs cd‐hit v.4.8.1 (Fu et al. 2012). We aligned sequences to our de novo assembly using bwa v.0.7.17 (Li and Durbin 2009) and generated associated .bam files with samtools v.1.15.1 (Danecek et al. 2021). Variant calling was performed in bcftools v.1.9 (Danecek et al. 2021) using the mpileup and multiallelic‐caller functions. We initially called SNPs based on a minimum mapping quality of 30, minimum base quality of 20, max depth of 250×, adjusted mapping quality of 50, and exclusion of indels. We further filtered SNPs in vcftools v.0.1.16 (Danecek et al. 2011) by excluding sites and individuals with missing data of > 30% and > 25%, respectively. To minimize paralogous and erroneous sites, we retained only biallelic loci with 20–30× depth of coverage and a minor allele frequency > 0.05. FASTQ files for each sample are available at the National Center for Biotechnology Information (NCBI) in the sequence read archive under BioProject PRJNA1328208 (accession numbers SAMN51307640‐SAMN51307946) and scripts for processing fastq files and downstream analyses are available at https://github.com/cdfwWildlifeGenetics/publications/tree/main/Ahrens%20et%20al.%202025%20Evol%20Appl%20nutria. Mitochondrial sequence data and VCF files are available on Dryad.

### 2.4 Genetic Analyses

#### 2.4.1 Mitochondrial DNA Analysis

We edited and aligned forward and reverse sequences of Cyt *b* and D‐loop fragments using Geneious Prime v.2023.1.2 (https://www.geneious.com) and re‐sequenced or discarded those with discrepancies or low‐quality scores. We concatenated the two Cyt *b* fragments and generated an alignment using the MAFFT algorithm (v7.490; Katoh et al. 2002; Katoh and Standley 2013) with default settings.

We analyzed Cyt *b* and D‐loop datasets separately. For our two mtDNA phylogenies, we used the painted tree‐rat ( *Callistomys pictus* ; GenBank accession no. KU892754; Fabre et al. 2017), the closest extant relative of nutria and the only mitochondrial sequence available, as the outgroup. We included a spatially representative subset of California nutria (*n* = 31), individuals from other USA localities (*n* = 40; Table S1), historical museum samples (*n* = 11; Table 2), and GenBank sequences from nutria within their native range and other invasive populations globally (Table S2). PartitionFinder v2.1.1 (Lanfear et al. 2017) was employed to determine the evolutionary model for both mtDNA loci, identifying GTR + G as the best fit. We then estimated relationships using raxml v.8.2.12 (Stamatakis 2006) using the ‘‐f a’ option, simultaneously determined the best‐scoring maximum likelihood (ML) tree, and conducted a rapid bootstrapping support analysis. A suitable number of bootstrap (bs) replicates (*n* = 1000) was determined by MRE‐based bootstopping criterion implemented in raxml using the ‘autoMRE’ option. Bootstraps ≥ 70 were considered indicative of significant relationships (Hillis and Bull 1993; Alfaro et al. 2003). To visualize haplotype diversity and frequency across geographic regions, we constructed median‐joining haplotype networks using PopART v. 1.7 (Leigh et al. 2015). Sequences for novel haplotypes were added to GenBank (Accession numbers PX240733‐35, Table S1).

#### 2.4.2 RADseq Phylogenetic Assembly

We estimated phylogenetic relationships with our RADseq dataset to further characterize genetic variation of nutria in the USA. RADseq data have demonstrated the ability to elucidate phylogenetic relationships across diverse timescales by generating large numbers of phylogenetically informative loci, all without requiring prior genomic information (Lemmon and Lemmon 2013; Ree and Hipp 2015; Leaché and Oaks 2017). Using a subset of contemporary California samples representing the spatial extent of the invaded area (*n* = 41) as well as samples from other states (*n* = 25, Table S1), we generated a concatenated assembly of loci using the de novo pipeline in ipyrad v.0.9.54 (Eaton and Overcast 2020). We parameterized ipyrad using default settings while excluding loci with ≥ 25% missing data. Following the same methodology as with the mtDNA dataset, we used raxml to estimate relationships (bootstrap replicates, *n* = 650).

#### 2.4.3 Genetic Population Structure

We sought to characterize nutria population genetic structure at multiple spatial scales, both within California and across the USA. First, to determine the genetic similarity of contemporary California nutria to other invasive populations in the USA, we performed principal coordinate analysis (PCoA) implemented in R v.4.3.1 (R Core Team 2023) and the package dartr v.2.9.7 (Gruber et al. 2018) on all SNP loci. To avoid biased results due to sample size, we selected a subset (*n* = 11) of nutria from California that was geographically representative of the invaded area. As an additional test, we partitioned nutria from outside California into ‘East’ (Louisiana, Maryland, Texas, Virginia) and ‘West’ (Oregon, Washington) groups and identified loci with alleles private to each. We then performed a second PCoA on the reduced set of loci for all samples. We further determined the number of private alleles California shared with each group using ‘gl.report.pa’ in dartr. To understand the genetic composition of the current California invasion and patterns of gene flow, we then analyzed the California dataset independently. Lastly, we performed a third PCoA for all loci using only samples from California.

We further examined population structure using two separate structure v.2.3.4 (Pritchard et al. 2000) analyses. First, we compared all East and West samples with a subset of California samples (*n* = 81) across all the SNP loci. We examined *K* = 2–5, running each replicate (*n* = 10) for 1 × 10^7^ Markov chain Monte Carlo iterations, with a burn‐in period of 25 × 10^4^. In the second analysis, we included all USA samples but limited the SNPs to the reduced set of loci (i.e., those with private alleles between East and West). Using the same parameters, we then ran structure at *K* = 2–3.

We calculated genetic differentiation between the core invaded area (the Grasslands Ecological Area, Merced NWR, Merced River, and San Joaquin River) and the leading edges of the invasion (Sheman Island in the north, Mendota WA in the south) using the Weir and Cockerham (1984) method of estimating pairwise *F*_ST_ performed in the R package hierfstat v.0.5‐11 (Goudet 2005; Goudet et al. 2022). We then calculated pairwise *F*_ST_ among all MUs, as well as pairwise conditional genetic distance (*cGD*) using the R package gstudio v.1.6.0 (Dyer 2014). Conditional genetic distance measures both direct and indirect connectivity due to differences in genetic covariation and may be more sensitive for landscape genetic modeling than traditional pairwise genetic distance metrics (Dyer et al. 2010).

#### 2.4.4 Genetic Diversity and Kinship

We calculated summary statistics within each MU to determine whether differences existed across the invaded area. We used the R package hierfstat v.0.5‐11 (Goudet 2005) to calculate expected heterozygosity (*H*_E_) and the inbreeding coefficient (*F*_IS_). We estimated pairwise kinship between all individuals using the king package (Manichaikul et al. 2010) implemented in plink 2.00 alpha v.2.3 (Chang et al. 2015). We then estimated mean kinship within each MU by calculating the average of all pairwise comparisons.

#### 2.4.5 Isolation‐By‐Distance and Migration Surface Modeling

Given the semi‐aquatic life history of nutria and potential sex differences in philopatry to natal sites (Lee et al. 2021), we tested multiple models of isolation‐by‐distance (IBD) at the individual level. We performed a Mantel test using the R package vegan (v.2.6‐4; Oksanen et al. 2024), correlating Euclidian distance between each sample location with individual‐based analysis of molecular variance (AMOVA) genetic distance calculated in gstudio (Dyer 2014). We also performed a separate Mantel test for each sex (males, *n* = 145; females, *n* = 113). As an alternative to Euclidean distance, we used ArcGIS Pro v.3.1.2 with the Network Analyst plugin to calculate an ecological distance in the form of pairwise ‘river’ distances between nutria. We used the National Hydrography Dataset maintained by the U.S. Geological Survey (https://nhd.usgs.gov/User_Resources_Overview.html) and included all natural water bodies as well as canals. We used the Origin–Destination (OD) Cost Matrix analysis in ArcGIS Pro to determine the shortest river distance between sample points constrained to follow such water features. A Mantel test was performed to assess the correlation between AMOVA genetic distances and the resulting river distances. We also constructed correlograms using the R package vegan to observe the Mantel *r* correlations across discrete distance classes.

We generated estimated effective migration surfaces (eems; Petkova et al. 2016) to characterize patterns of dispersal among MUs in a spatially explicit fashion. By evaluating deviations from IBD among demes (*n* = 600) using a stepping‐stone model across a predefined region, eems infers corridors that may facilitate or impede connectivity across the landscape (reviewed in House and Hahn 2018; Petkova et al. 2016). We defined our study region in ArcGIS Pro by creating a polygon buffer of approximately 20% the width of the sampled area to reduce edge effects.

Pairwise genetic distances between individuals were calculated using AMOVA in gstudio. We then conducted preliminary analyses to ensure proposal rates were not accepted too infrequently (< 10%) or too often (> 40%). For our final analysis, we ran three independent chains for 1 × 10^7^ iterations with an initial burn‐in of 1 × 10^6^, sampling every 5000 iterations. Convergence and model parameters were evaluated using the R package reemsplots2, and we visualized plots with the ‘make_eems_plots’ function.

## 3 Results

### 3.1 Mitochondrial DNA Analyses

**FIGURE 3:**
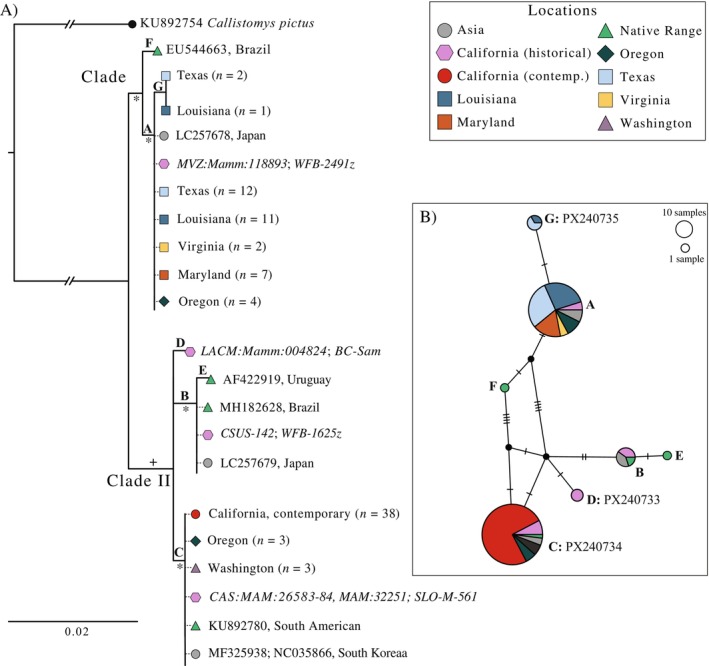
Mitochondrial DNA relationships for cytochrome *b* locus (555 bp, Table 3) examining the current California invasion (*n* = 38), historical samples from California fur farms (*n* = 11), other USA invasive populations (*n* = 46), and GenBank sequences (*n* = 8) from nutria populations globally (Table S2). (A) raxml phylogeny with 1000 bootstraps was performed, branch support ≥ 80 indicated by + and ≥ 90 indicated by *. Haplotype names are listed above each branch and follow the naming convention of Kawamura et al. (2018). (B) Median‐joining network depicting haplotype frequencies by location. Black circles indicate unsampled haplotypes, and hashmarks represent mutational steps. Haplotype names are indicated and novel haplotypes with their corresponding GenBank accession number are also provided.

The concatenated 555 bp Cyt *b* sequence resulted in 11 variable sites and seven haplotypes across all samples (Table S1). All contemporary California nutria shared a single haplotype. This haplotype was also present in Washington and central Oregon, as well as GenBank sequences for invasive nutria in South Korea and native nutria from South America (Figure 3; Table S2). This haplotype formed a clade (Clade II, bs = 80) with GenBank sequences for invasives in Japan and native nutria from Brazil and Uruguay. A highly supported second clade (Clade I, bs = 98) was formed primarily by sequences from the eastern USA (East), nutria from northern Oregon, and GenBank sequences for invasives from Japan and native nutria from Brazil. Ten historical samples sequenced successfully for both Cyt *b* fragments, resulting in four haplotypes spanning both clades and seven variable sites, clearly indicating California fur farm nutria had greater genetic diversity prior to eradication in the 1970s. Further, the historical samples broadly showed genetic affinity with both invasive and native populations globally. Four historical samples shared the single haplotype found in contemporary California nutria (Clade II; Figure 3), while two others fell within Clade I, including a shared haplotype with invasives from Japan as well as native nutria from Brazil. The remaining historical samples fell within Clade I, sharing haplotypes with invasive nutria from the East and Japan.

The 514 bp D‐loop sequence resulted in 16 variable sites and four unique haplotypes (Figure S1). All four haplotypes were previously observed in a native nutria population in Argentina (Ibañez et al. 2021). Individuals with a common Cyt *b* haplotype also shared a D‐loop haplotype. Given the broad geographic distribution of our samples, the low haplotype diversity observed was unexpected, considering that Ibañez et al. (2021) described 28 unique D‐loop haplotypes from a single watershed within the native range. Our sequencing success for the historical samples was lower, given the larger size of our target D‐loop fragment, but only slightly (*n* = 9). Bootstrap support for the raxml phylogeny was categorically poor, with all branches receiving < 70 support (Figure S1). All contemporary California nutria shared a single haplotype which was also present in both Oregon and Washington, as well as four historical samples from fur‐farmed nutria. This single haplotype formed a poorly supported clade (Clade II, nested within Clade I), with two additional haplotypes recovered from historical samples and GenBank sequences for invasives from South Korea and several other native haplotypes from Argentina. A fourth haplotype was shared among invasive nutria in the East, a subset of Oregon samples, two historical samples, and natives. Taken together, the mtDNA loci we examined revealed no diversity within the contemporary California invasion.

### 3.2 SNP Genotyping

After filtering and calling, we retained 6809 SNPs from our RADseq dataset with a mean depth of coverage of 24.5× (SD = 2.8×). Average missing data across all 307 individuals was 0.49% (SD = 1.2%), and the maximum missingness for a given individual was < 12%. Replicate genotypes generated across lanes of Illumina sequencing had high pairwise KING‐robust kinship coefficients (> 0.4), indicating minimal batch effects across lanes.

### 3.3 RADseq Phylogenetic Assembly

**FIGURE 4:**
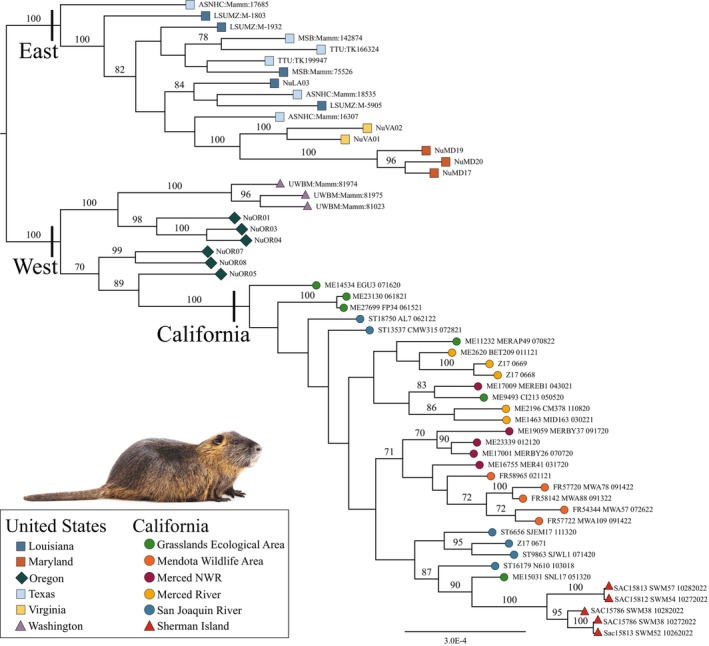
Maximum likelihood‐based (raxml) nuclear (RADseq) phylogeny based on 7,274,362 sites and 650 bootstrap replicates, with values indicated for branches with support of ≥ 70. Strongly supported monophyletic clades separate East from West and the current California invasion. The closest association of contemporary California nutria is with samples from central Oregon, while northern Oregon samples group with Washington nutria.

After quality filtering, the resulting RADseq assembly was 7,274,362 bp in length, consisting of 57,090 loci, 28,927 of which were parsimony‐informative, with 17.3% missing sites. We recovered two broad geographic clades representing the East (bs = 100) and West (bs = 100) USA (Figure 4). Within these clades, finer‐scale relationships were recovered with bootstrap support > 70. Among East samples, Virginia and Maryland were sister to one another, forming monophyletic clades. There was limited support for specific relationships among samples from Texas and Louisiana, and neither region was monophyletic. In the West, Washington and California both formed strongly supported monophyletic clades, while Oregon nutria were polyphyletic. Samples from northern Oregon were sister to the Washington clade, with central Oregon more closely related to the California clade.

Within the California clade, relationships among MUs showed poor bootstrap support and high paraphyly. The single exception was Sherman Island at the northern leading edge of the invasion (bs = 100). This group was most closely related (bs = 87) to samples from both the Grasslands Ecological Area and San Joaquin River. Nutria from Mendota WA, at the southern leading edge of the invasion, formed a poorly supported (bs = 30) clade that was most closely related to a subset of individuals from Merced NWR (bs = 71).

### 3.4 Genetic Population Structure

**FIGURE 5:**
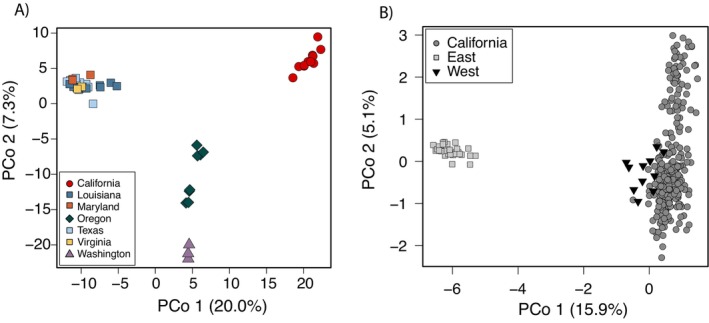
Principal coordinate analyses (PCoA) for (A) nutria from throughout the USA, including a random subset from California (*n* = 11) that is proportional to the sample sizes for other USA populations and (B) analysis of 403 SNP loci which were identified as containing private alleles within the East (Louisiana, Maryland, Texas and Virginia; 87 loci) or West (Oregon and Washington; 316 loci) regions of the USA. Following identification of private alleles, California samples were added back to the dataset to determine the relationship between California and other USA nutria populations.

PCoA revealed strong genetic differentiation between the East, West, and California populations (Figure 5A) along principal coordinate axis 1 (PCo1; 20% variance explained). Interestingly, there was minimal genetic differentiation among populations in the East (Louisiana, Maryland, Texas, Virginia), despite the large geographic distribution of these samples. Conversely, clear differentiation among samples from Washington and northern and central Oregon was evident along PCo2 (7.3% variance explained). Contemporary California nutria formed a distinct cluster with no evidence of admixture with other invasive USA populations. Results from structure analyses also supported that California nutria represented a distinct population, though at *K* = 2, California shared ancestry with populations in the West (Oregon and Washington). Nutria from the eastern USA were uniformly differentiated for *K* = 2–5 (Figure S2A).

We identified 403 SNP loci with alleles private to either the East or West nutria populations. Among these, 87 possessed alleles private to the East and 316 loci had alleles private to the West, with no fixed allele differences between the two (Table 4). Comparison of contemporary California nutria to East and West populations for the reduced SNP dataset revealed that they shared 261 private alleles with the West and 142 with the East. The PCoA with the reduced SNP dataset indicated a close affinity between California and the West (Figure 5B), with high overlap along PCo1 (15.9% of variance explained). structure results for *K* = 2 and 3 also supported a closer association between the West and California (Figure S2B).

**TABLE 4:** Filtered SNP dataset with 403 loci selected for alleles private to either the East or West regions of the USA. The numbers of alleles private to each group as well as shared between groups are indicated.

Pop 1	Pop 2	Private to pop 1	Private to pop 2	Total shared
East	West	87	316	0
East	California	47	214	142
California	West	40	102	261

**FIGURE 6:**
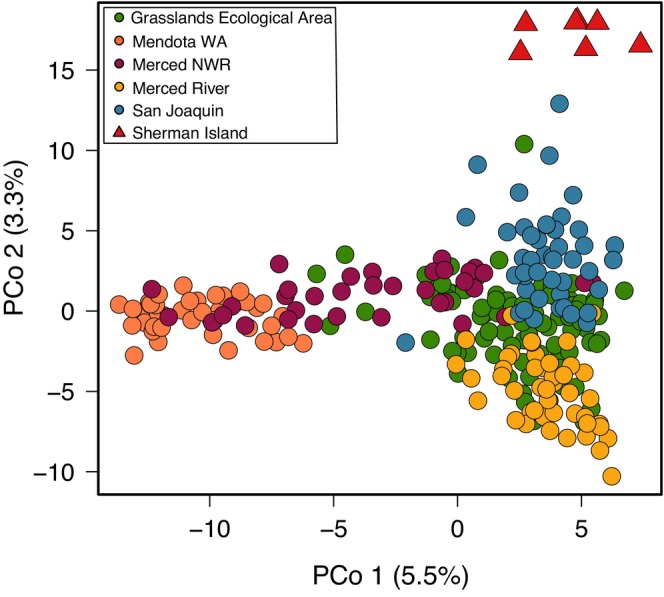
Principal coordinate analyses (PCoA) for 267 contemporary California nutria.

PCoA of contemporary California nutria indicated a lack of discrete clustering (Figure 6). However, nutria from geographically adjacent MUs largely overlapped in principle coordinate space, particularly in the core of the invaded area, suggesting few barriers to gene flow. The leading edges of the invasion displayed the greatest differentiation. Sherman Island at the northern leading edge formed a distinct cluster along PCo2 (3.3% variance explained), while Mendota WA at the southern leading edge differentiated along PCo1 (5.5% variance explained) while showing overlap with Merced NWR. Our structure results suggested minimal population structure among the MUs, with Sherman Island and Mendota WA forming distinct genetic clusters at *K* = 4 and *K* = 5, respectively (Figure S2A).

Pairwise *F*_ST_ calculations between the core invaded area (the San Joaquin River, Merced River, Grasslands Ecological Area, and Merced NWR) and the leading edges of the invasion suggested that Sherman Island was strongly differentiated (*F*_ST_ = 0.16), while Mendota WA was moderately differentiated (*F*_ST_ = 0.07). Pairwise *F*_ST_ values were low (≤ 0.05) among MUs in the core invaded area, indicating high levels of connectivity and gene flow. At the northern leading edge of the invasion, high *F*_ST_ and *cGD* values further supported Sherman Island as substantially differentiated from other MUs, including the geographically nearest MU, the San Joaquin River (*F*_ST_ = 0.16, *cGD* = 2.57; Table 1). At the southern leading edge of the invasion, the lowest pairwise *F*_ST_ and *cGD* values observed between Mendota WA and another MU were with the Merced NWR (*F*_ST_ = 0.06, *cGD* = 0.84).

### 3.5 Genetic Diversity and Kinship

Differences in genetic diversity statistics among nutria in the core invaded area were minimal, with the most evident differences among MUs at the northern and southern leading edges of the invasion (Table 1). Average expected heterozygosity within the core invaded area was moderate (*H*_E_ = 0.32), with lower levels observed for Sherman Island (*H*_E_ = 0.20) and Mendota WA (*H*_E_ = 0.28). Similarly, measures of *F*_IS_ were comparable across all MUs with no substantial evidence of inbreeding (mean *F*_IS_ = −0.033), apart from Sherman Island, where *F*_IS_ = −0.20. Mean kinship across the entire invaded area was low (−0.053) and within 5 of the 6 MUs ranged from −0.086 to 0.02, suggesting nutria were largely unrelated. Sherman Island was the exception, where mean kinship was high (0.15) and a family group of two related adult females, one adult male, and combinations of their four offspring were identified.

### 3.6 IBD and EEMS

**FIGURE 7:**
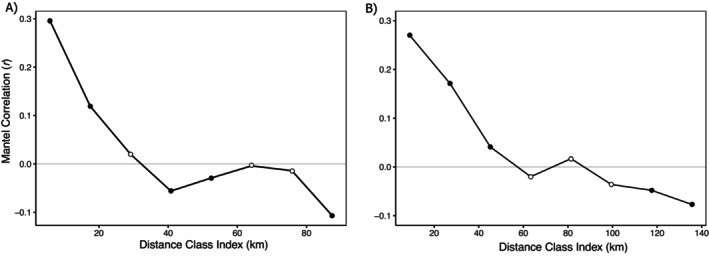
Mantel correlograms using individual‐based pairwise comparisons of AMOVA genetic distance with (A) Euclidean distance across 11.7 km distance classes (overall IBD *r* = 0.379; *P <* 0.001) and (B) river distance based on the shortest artificial and/or natural waterway distance using 18.1 km distance classes (overall IBD *r* = 0.42; *P <* 0.001). Black circles indicate significant Mantel *r* statistics.

The Mantel test for IBD using pairwise Euclidean distance versus AMOVA genetic distance showed a strong correlation (Mantel *r* = 0.38, *P* < 0.001). Perhaps unsurprisingly given the semi‐aquatic life history of nutria, the Mantel test using ‘river’ distance showed a stronger correlation with genetic distance (Mantel *r* = 0.42, *P* < 0.001). Sex‐specific Mantel tests confirmed that river distance had a stronger correlation with genetic distance than Euclidean distance for both males and females, with a higher test statistic observed among females (Mantel *r* = 0.48, *P* < 0.001; vs. male Mantel *r* = 0.39, *P* < 0.001). Correlograms obtained using both Euclidean distance (Figure 7A) and river distance (Figure 7B) indicated that Mantel test statistics remained significant over greater distance classes for river distance (> 40 km).

**FIGURE 8:**
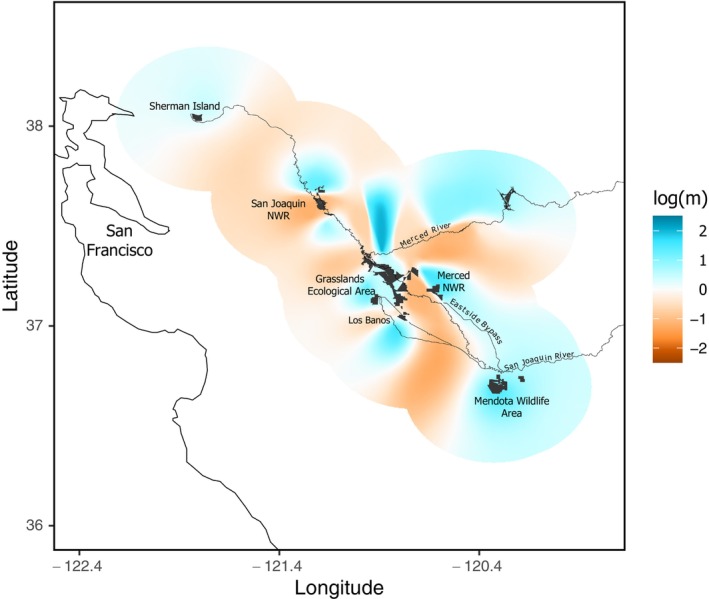
Estimated effective migration surface (eems) modeling representing deviations from isolation‐by‐distance and showing inferred corridors/barriers to dispersal and range expansion when 600 demes were applied to the total study area. The study area included in the analysis was generated with a 20% polygon buffer (shaded area) around sampling points. Black shaded polygons represent state/federal managed wetland areas.

The eems analysis indicated significant spatial variation in gene flow throughout the study area (Figure 8). Generally, gene flow exceeded the predictions of IBD in areas coincident with large managed wetlands (e.g., Grasslands Ecological Area, Merced NWR, Mendota WA, etc.). Conversely, areas between managed wetlands, which frequently coincided with major river corridors, exhibited less gene flow than predicted by IBD. The eems analysis indicated high gene flow between the Merced NWR and the southern leading edge of the invasion (Mendota WA) to the southwest around the Eastside Bypass canal.

## 4 Discussion

We characterized the genetic population structure of invasive nutria across the USA, while focusing on the recently identified invasion of the Central Valley and Sacramento‐San Joaquin Delta of California. Our RADseq analyses identified well‐supported East and West lineages (Figure 5), with California sharing the closest relationship with central Oregon (Figure 4). Mitochondrial DNA sequencing resulted in relatively few Cyt *b* haplotypes, and phylogenetic analyses mirrored the RADseq data, with haplotypes largely conforming to the East and West USA clades and a shared haplotype between California, Oregon, and Washington (Figure 3). The absence of mtDNA diversity within the contemporary California invasion was in clear contrast with historical samples from fur‐farmed nutria (1936–1960), which had higher diversity and shared haplotypes with nutria from multiple global native and invasive populations, including the eastern USA and Japan. There was minimal population structure present within contemporary California nutria, with the most notable differentiation at the leading edges of the invasion. Thus, our data supported considering the Central Valley a single eradication unit. We further demonstrated that genetic tools can assist in identifying dispersal pathways in invasive species, even in the case of a recent invasion followed by rapid range expansion, providing insight for managers as they direct eradication efforts.

### 4.1 Low Genetic Diversity Suggests a Single, Recent Founder Event

Our study is the first to address relationships among invasive nutria populations across the USA, providing greater context for the invasion history of California. We compared mtDNA sequences from contemporary California nutria to invasions in numerous other states, as well as sequences archived on GenBank from both native and invasive populations globally. Interestingly, we observed relatively little Cyt *b* haplotype diversity throughout the USA, finding only five haplotypes among 95 samples (Figure 3). Three of these haplotypes had large geographic distributions and were shared among nutria on multiple continents. For example, the single haplotype found in contemporary California nutria was also found in Oregon, Washington, South Korea, South America, and historical California fur‐farmed individuals. This pattern of low haplotype diversity and broad geographic distribution is consistent with the known global invasion dynamics of nutria, namely farm establishment via the importation of a relatively small number of founders and a subsequent bottleneck following the escape or release of farmed individuals. Our results are similar to those of Kawamura et al. (2018), who found only two Cyt *b* haplotypes in a ~70‐year‐old invasion in Japan, originating from a single importation of 150 nutria. This is in stark contrast to the large haplotype diversity observed within a small portion of the native range (Ibañez et al. 2021, 2024). Those authors found 28 D‐loop haplotypes within a single watershed in Buenos Aires Province, Argentina, whereas our D‐loop sequencing found only four of these previously described haplotypes distributed across the entire USA (Figure S1). This pattern is illustrative of the ‘invasion paradox,’ wherein genetic bottlenecks typical of founder events in invasive populations do not necessarily inhibit invasion success (Allendorf and Lundquist 2003; Dlugosch et al. 2015; Estoup et al. 2016; Heckwolf et al. 2021; Buchholz et al. 2023). Future genetic studies of invasive nutria should build on the Cyt *b* and D‐loop datasets to test whether such low diversity is consistent across invasions while simultaneously expanding the means for tracking nutria lineages and the source of new invasions globally.

While we found only a single haplotype at both Cyt *b* and D‐loop loci among 31 contemporary California nutria, our results also indicated there was higher diversity in California prior to eradication in the 1970s. Of the 11 historical fur farm samples sequenced at the Cyt *b* locus, 4 different haplotypes were present, one of which matched the contemporary haplotype. Fur farming in California occurred between the 1930s and 1960s, after which feral populations formed from escapees of numerous farms raising one or more lineages. As a result, historical California samples included multiple mtDNA haplotypes that are shared with other global invasive populations. In the absence of detailed importation records, genetic data may be the only means of assessing the extent to which California fur farms represented mixed‐source stock.

The single haplotype found in contemporary samples is consistent with a recent invasion propagated from a small number of individuals (i.e., a single maternal lineage). Because the contemporary haplotype was also present in historical fur‐farmed nutria, the possibility of a recent expansion of a remnant population cannot be excluded. Following introduction, some invasive species will remain limited to a small area for several years (i.e., lag phase) before rapidly expanding; however, this phenomenon is largely known in invasive plants (Mack et al. 2000; Robeck et al. 2024). Given the mild climate of California combined with the reproductive capacity, short generation time, and dispersal ability of these invasive rodents, it is unlikely that a relic population could have gone undetected since the previous eradication. However, this possibility cannot be refuted by the mtDNA data alone.

Prior to this study, the contemporary invasion was hypothesized to have originated from a single human introduction within the Grasslands Ecological Area or Merced River watershed (Figure 2). Our nuclear RADseq and mtDNA phylogenies, which included broad geographic sampling, allowed for inference regarding the potential source population, a level of analysis not attempted in previous genetic studies of nutria. The SNP dataset filtered for private alleles within the East and West USA populations showed a clear relationship between the West and California (Figure 5B; Table 4), and the nuclear RADseq phylogeny (Figure 4) indicated contemporary California nutria are most similar to those in central Oregon, suggesting that the founder population either originated within this region or was derived from a common source stock. This finding was further supported by the mtDNA phylogenies, as the single contemporary California haplotype was shared with samples from central Oregon. Nutria were first imported to Oregon for fur farming in 1937, with feral populations officially recorded as early as 1941 (Larrison 1943). Since then, a well‐established population has persisted without a formal eradication program in place. It is clear from our results that the global importation of nutria resulted in a broad distribution of certain common haplotypes, suggesting that identification of the source of new nutria invasions using mtDNA alone may prove challenging. The use of both nuclear SNP and mitochondrial markers provides different modes of inference when studying the evolutionary history of a species (Rubinoff and Holland 2005; Tollefsrud et al. 2009; Eytan and Hellberg 2010; Jiang et al. 2016), which is particularly useful for investigating the origin and dynamics of invasive species. We recommend future studies of nutria invasion genetics use both methods.

### 4.2 Gene Flow, Population Structure, Dispersal Pathways, and Designation of Eradication Units

Our PCoA results suggest high gene flow and minimal population structure throughout the core invaded area of the Central Valley (Figure 6). Minimal genetic structure was also apparent from the nuclear RADseq phylogeny, as indicated by low bootstrap support for branching among the California samples (Figure 4), while pairwise *F*_ST_ estimates among management areas in the core invaded area (excluding the leading edges of the invasion) were low and did not exceed 0.06 (Table 1). This general lack of population structure may be the result of an initial founder event leading to reduced genetic variation. This can make genetic structure challenging to detect, as has previously been noted in nutria (Klima and Travis 2012). Second, if the point of invasion was localized, followed by rapid expansion from this single source, there would have been little time for genetic differentiation to occur (Drygala et al. 2016). Lastly, it is unclear whether sufficient generation time has lapsed to detect barriers to gene flow. While landscape features that impede dispersal can lead to moderate levels of differentiation over time (Landguth et al. 2010), detection of genetic structure may greatly exceed time scales relevant to statewide eradication efforts.

Population structure was more apparent at the northern and southern leading edges of the invasion. Nutria from Sherman Island, and to a lesser extent Mendota WA, showed more cluster separation in the PCoA (Figure 6), with the former showing high genetic differentiation (pairwise *F*_ST_ between 0.16 and 0.26, Table 1) from other areas. This differentiation is likely the result of range expansion via colonization of unoccupied habitats by a small number of founders, followed by a period of close inbreeding. This scenario is supported by the high level of kinship at Sherman Island, the most recently colonized area at the time of sampling, where we identified a family group. We found higher kinship and lower heterozygosity at the leading edges of the invasion, which typifies genetic bottlenecks caused by founder events (Wright 1931; Nei et al. 1975). The high kinship estimates observed at Sherman Island also conform with the known mating system and social structure of nutria: polygyny, with breeding females forming kin groups with their juvenile offspring (Guichón et al. 2003; Túnez et al. 2009).

Our SNP results indicate a pattern of IBD, as seen in both PCoA and Mantel tests. The PCoA revealed an overlap in ordination space among geographically adjacent management areas, suggesting that gene flow occurs in a stepping‐stone fashion, where individuals are most genetically similar to the nearest‐neighboring area (Figure 6). The northwest–southeast differentiation across the invaded area represented < 6% of genetic variance (PCo1, Figure 6). Mantel tests indicated Euclidean distance explained up to ~14% of the nuclear genetic variance. This contrasts sharply with the absence of IBD found in nutria from southern Louisiana (Klima and Travis 2012), where no significant increase in genetic differentiation with increasing geographic distance was observed (Mantel *r* = 0.12, *P* = 0.3) among eight watersheds that had been invaded for ~75 years. However, Kawamura et al. (2018) found strong IBD (*r* ^2^ = 0.56, *P* < 0.001) among nine river drainages in Japan that had been invaded for a similar amount of time. It is important to note that the study area in Louisiana was significantly larger than both that of Kawamura et al. and our own, suggesting the detection of population structure by IBD may be spatial scale‐dependent. Future studies, particularly those over large areas, should make similar use of methods that evaluate IBD over distance classes (i.e., correlograms). Alternatively, long‐term invasions in highly mobile species with limited dispersal barriers may attain a higher degree of panmixia, characterized by extensive gene flow throughout the invaded area (Fischer et al. 2017), with IBD representing a short‐term phenomenon characteristic of recent invasions. Future studies of nutria invasions should pay close attention to both the spatial and temporal patterns of IBD to better understand invasion dynamics.

Understanding modes of dispersal is key to addressing range expansion in invasive species. Given the semi‐aquatic life history of nutria, we also used ‘river’ distance as an alternative to Euclidean distance‐based Mantel tests. River distance yielded a stronger, significant correlation (Mantel *r* = 0.42, *P* < 0.001) with genetic distance. Further, comparison of correlograms of both Euclidean and river distance indicated significant Mantel correlations were supported at larger distance classes when river distance was used; up to 45 km versus only 18 km in the case of Euclidean distance (Figure 7). Our results provide insight into the importance of riparian corridors and canals for nutria movement; however, this should not be assumed in other invaded areas outside of California or with other invasive semi‐aquatic mammal species. For example, Zalewski et al. (2009) evaluated IBD using both Euclidean and river distances for invasive American mink (*Neogale vison*) in Scotland and found Euclidean distance resulted in a higher, significant Mantel *r*. Future landscape genetic studies of nutria should compare Euclidean and ecological distances when testing patterns of IBD, landscape resistance, and gene flow.

The power of coupling high‐resolution SNP data with spatially explicit migration surface modeling allowed us to identify features influencing dispersal pathways in the complex landscape mosaic of the Central Valley. The eems analysis suggested gene flow was mediated by habitat type (Figure 8). The resulting surface indicated that gene flow within management areas, often representing large federal or state protected wetlands, was higher than predicted by IBD. Conversely, gene flow between protected wetland areas was often less than predicted by IBD. This pattern is consistent with a scenario where nutria rapidly colonize areas of emergent wetland habitat, while experiencing some landscape resistance to gene flow between habitat patches. This points to a period of exploratory dispersal through marginal habitat preceding the colonization of new wetland areas. The eems analysis further suggested that riparian corridors and certain canals (i.e., Eastside Bypass) are important for nutria dispersal in the Central Valley and were corroborated by the Mantel tests (Figure 7). Such drainage‐based dispersal patterns of nutria have been reported previously in the USA (Evans 1970) and South Korea (Hong et al. 2015).

### 4.3 Management Implications

The current California nutria invasion is most genetically similar to Oregon, which has implications for biosecurity and invasive species prevention strategies. Nutria are classified as an A‐rated pest by the California Department of Food and Agriculture (CDFA) and a Restricted Live Animal by CDFW (California Code of Regulations, Title 14, section 671), indicating that they are of known economic and environmental detriment, are prohibited from entering the state, and warrant regulatory enforcement and rapid response. As a first line of defense, CDFA operates 16 vehicle inspection stations at entry points into California. Despite these efforts, it is possible that the importation of nutria occurred through an alternate route into the state, gaps in surveillance, or during station closures due to seasonal timing or staffing limitations. The reintroduction of nutria into California highlights the value of investing in increased biosecurity measures, as prevention is consistently a far more cost‐effective approach to invasive species management than long‐term eradication or control efforts. Our data support the recommendation to consistently re‐evaluate state border protection practices and their efficacy in preventing further introductions of ecologically and economically detrimental invasive species, including invasive vertebrates.

Eradication efforts require cooperation among federal, state, and private entities; however, there are multiple constraints complicating nutria eradication in California. One is the extensive hydrologic connectivity across the region, including a complex network of agricultural canals, bypasses, wetland habitats, and natural water features which provide a multitude of dispersal pathways for nutria. Unlike coastal systems (i.e., Chesapeake Bay or southern Louisiana) where the open ocean limits the spread of nutria in at least one direction, California's Central Valley has few complete barriers to movement, allowing multidirectional dispersal. Another constraint is that a large proportion of the invaded area is private land that is inaccessible for CDFW management activities. While the CDFA Inspection Services Division has regulatory authority to conduct administrative searches for A‐rated pests on private property, CDFW has no comparable authority and relies solely on voluntary landowner cooperation. A third constraint involves the trapping of wetland areas important for either waterfowl hunting or water bird breeding, and as such are only accessible outside of the hunting season or restricted nesting periods. Such access limitations impact the continuity of eradication work, resulting in as little as 2–3 months of access per year in some cases. The combination of these time‐bound access and land ownership issues hinders sustained trapping, monitoring, and adaptive response, including implementation of the dispersal corridor‐based strategy supported by our data.

The eems analysis, *F*_ST_ and *cGD* values, and PCoA all indicated high connectivity between the Merced NWR and the southern leading edge of the invasion in Mendota WA (Figure 8). These areas are primarily connected by both the San Joaquin River and the Eastside Bypass canal, making either feature a potential corridor for nutria dispersal, though the significance of the latter is unclear. The Eastside Bypass is a wide, earth‐lined canal engineered for flood control and water conveyance. Because it is only seasonally inundated, it has minimal riparian cover or wetland vegetation. However, during high‐flow conditions in the San Joaquin River, it may serve as an easier dispersal route for upstream movement of nutria to Mendota WA. Use of the Eastside Bypass may also be highly limited by season and drought conditions, as seasonal inundation may be required, limiting upstream movements to winter months. During dry years when little to no water is present in the bypass, dispersal may not be possible. The genetic similarity between Mendota WA and Merced NWR lies in contrast to the larger genetic differentiation of Mendota WA from Grasslands Ecological Area, suggesting that the landscape south of the Grasslands Ecological Area represents a less significant corridor for southern expansion of nutria.

Despite eradication efforts, the invaded area has spread both upstream and downstream throughout the San Joaquin River drainage since the time of our genetic sampling, including the Sacramento‐San Joaquin Delta, Suisun Marsh, the Fresno River, and headwaters of the San Joaquin River. Our data show that once nutria colonize patches of emergent wetland, they encounter little resistance to expansion. In such cases, eradication efforts must be larger in scale, simultaneously targeting the entire invaded area as well as potential dispersal corridors (Mack et al. 2000; Anderson et al. 2022). Unless dispersal between MUs can be prevented, the Central Valley and Delta should be treated as a single eradication unit. For comparison, in the United Kingdom, a 7000 km^2^ area saw successful eradication of nutria over the course of 50 years (Gosling and Baker 1989; Carter and Leonard 2002). In Chesapeake Bay, USA, trapping of ~14,000 nutria from ~1000 km^2^ of wetland habitat dispersed throughout a 17,000 km^2^ project area achieved eradication after 13 years (Pepper et al. 2018; Anderson et al. 2022). By comparison, as of 2025, the known area of invasion in California spans over 5000 km^2^ of habitat dispersed throughout a 33,000 km^2^ project area. Over 6000 nutria have been culled to date, a number of take that has nearly doubled since the conclusion of this genetic sampling in 2022, despite consistent effort in removal. Successful eradication efforts suggest removal of nutria from California may represent a multi‐decade investment of resources, barring new innovations in eradication methods.

More broadly, the global extent of nutria invasions necessitates the use of commensurate genetic data that allow researchers to monitor population trends and accurately identify the sources of new invasions. This study demonstrates the utility of comparing nutria genetics at regional, continental, and global scales, and future studies should build on the foundation of population and phylogenetics presented here. This dataset provides a basis for the scientific management of global nutria populations and the protection of species and habitats imperiled by this highly invasive species.

## Conflicts of Interest

The authors declare no conflicts of interest.

## Supporting information


**Figure S1:** Contemporary USA nutria, California historical samples (*n* = 9; see Table 2), and native range nutria from Ibañez et al. (2021; GenBank Accession Nos. MZ153290‐MZ153317) sequenced at a 514 bp portion of the mitochondrial DNA D‐loop locus. (A) Phylogenetic tree (raxml) analysis with 1000 bootstraps where support was < 70 for all branches. Note that Clade II is nested within Clade I. (B) Haplotype network denoting frequency of haplotypes (circle size) and region of origin. Black circles indicate unsampled haplotypes, and hashmarks represent mutational steps.
**Figure S2:** structure bar plots for (A) the full 6809 SNP dataset at *K* = 2 through *K* = 5 and (B) the 403 SNP loci filtered for private alleles in East versus West at *K* = 2 and *K* = 3. All plots were generated with 10 iterations for each *K*, a burn‐in period of 25 × 10^4^, and 1 × 10^7^ MCMC.

**Table S1:** Supporting data for contemporary nutria samples included in the study. If samples were anlayzed at the mitochondrial D‐loop and Cyt *b* loci, corresponding haplotypes are provided. Novel Cyt *b* haplotypes C, D, and G were uploaded to NCBI GenBank under acccesion numbers PX240733‐35. Fastq files can be found on NCBI Sequence Read Archive under BioProject 1328208. Some analyses included only a subset of the total dataset as indicated in columns H‐L. *denotes samples which failed RADseq filtering and were analyzed at mtDNA loci only.
**Table S2:** GenBank Accession Numbers for previously published mitochondrial DNA sequences included in the study and which loci (D‐loop, Cyt *b*) were examined. KU892754* represents the sequence used as the outgroup, the painted tree‐rat (*Callistomys pictus*), while all other sequences are nutria (*Myocastor coypus*).

## Data Availability

Novel mitochondrial DNA sequences were uploaded to the NCBI GenBank Nucleotide Database (accession numbers: PX240733, PX240734, and PX240735). Illumina FASTQ files are available on the NCBI Sequence Read Archive (SRA) under BioProject PRJNA1328208 (accession numbers SAMN51307640–SAMN51307946). Scripts and R code can be accessed at: https://github.com/cdfwWildlifeGenetics/publications/tree/main/Ahrens%20et%20al.%202025%20Evol%20Appl%20nutria. Mitochondrial sequence data and VCF files can be found on Dryad: https://doi.org/10.5061/dryad.f1vhhmh92. All other data can be found in Supporting Information.
