# Supplementary material for: Population Genomic Insights Into Recent Nutria (Myocastor coypus) Invasion Dynamics
Source: Evol Appl. 2025 Nov 24;18(11):e70168. doi: 10.1111/eva.70168 (PMC12643119; doi:10.1111/eva.70168)
Supplement: Supplementary file 1 — Figure S1: Contemporary USA nutria, California historical samples (n = 9; see Table 2), and native range nutria from Ibañez et al. (2021; GenBank Accession Nos. MZ153290‐MZ153317) sequenced at a 514 bp portion of the mitochondrial DNA D‐loop locus. (A) Phylogenetic tree (raxml) analysis with 1000 bootstraps where support was < 70 for all branches. Note that Clade II is nested within Clade I. (B) Haplotype network denoting frequency of haplotypes (circle size) and region of origin. Black circles indicate unsampled haplotypes, and hashmarks represent mutational steps. Figure S2: structure bar plots for (A) the full 6809 SNP dataset at K = 2 through K = 5 and (B) the 403 SNP loci filtered for private alleles in East versus West at K = 2 and K = 3. All plots were generated with 10 iterations for each K, a burn‐in period of 25 × 104, and 1 × 10^7^ MCMC. [file EVA-18-e70168-s002.docx]

**Supplementary Information – Figures**

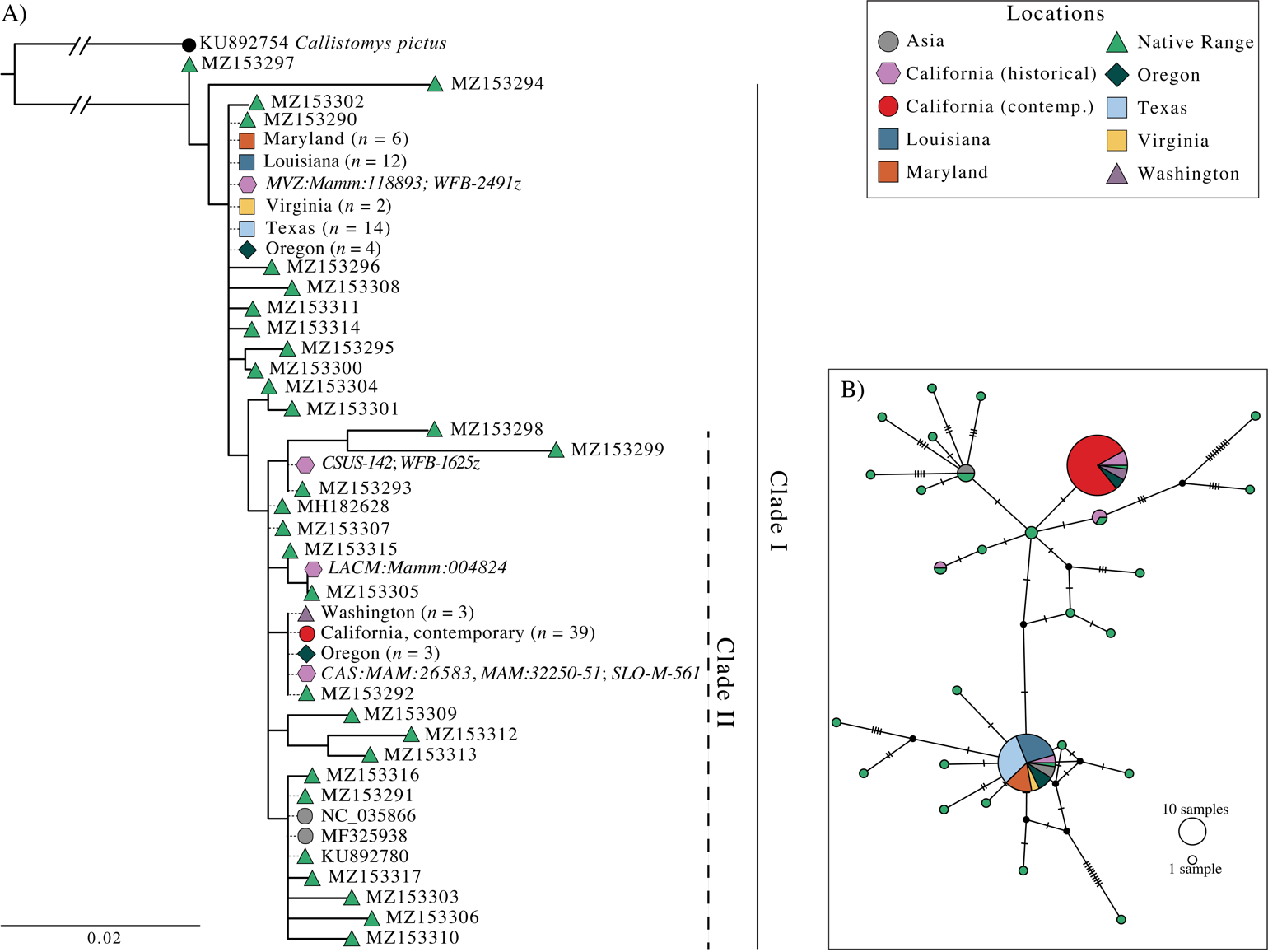


**Fig. S1** Contemporary USA nutria, California historical samples (*n* = 9; see Table 2), and native range nutria from Ibañez et al. (2021; GenBank Accession Nos. MZ153290-MZ153317) sequenced at a 514 bp portion of the mitochondrial DNA D-loop locus. **A)** Phylogenetic tree (raxml) analysis with 1000 bootstraps where support was <70 for all branches. Note that Clade II is nested within Clade I. **B)** Haplotype network denoting frequency of haplotypes (circle size) and region of origin. Black circles indicate unsampled haplotypes and hashmarks represent mutational steps.


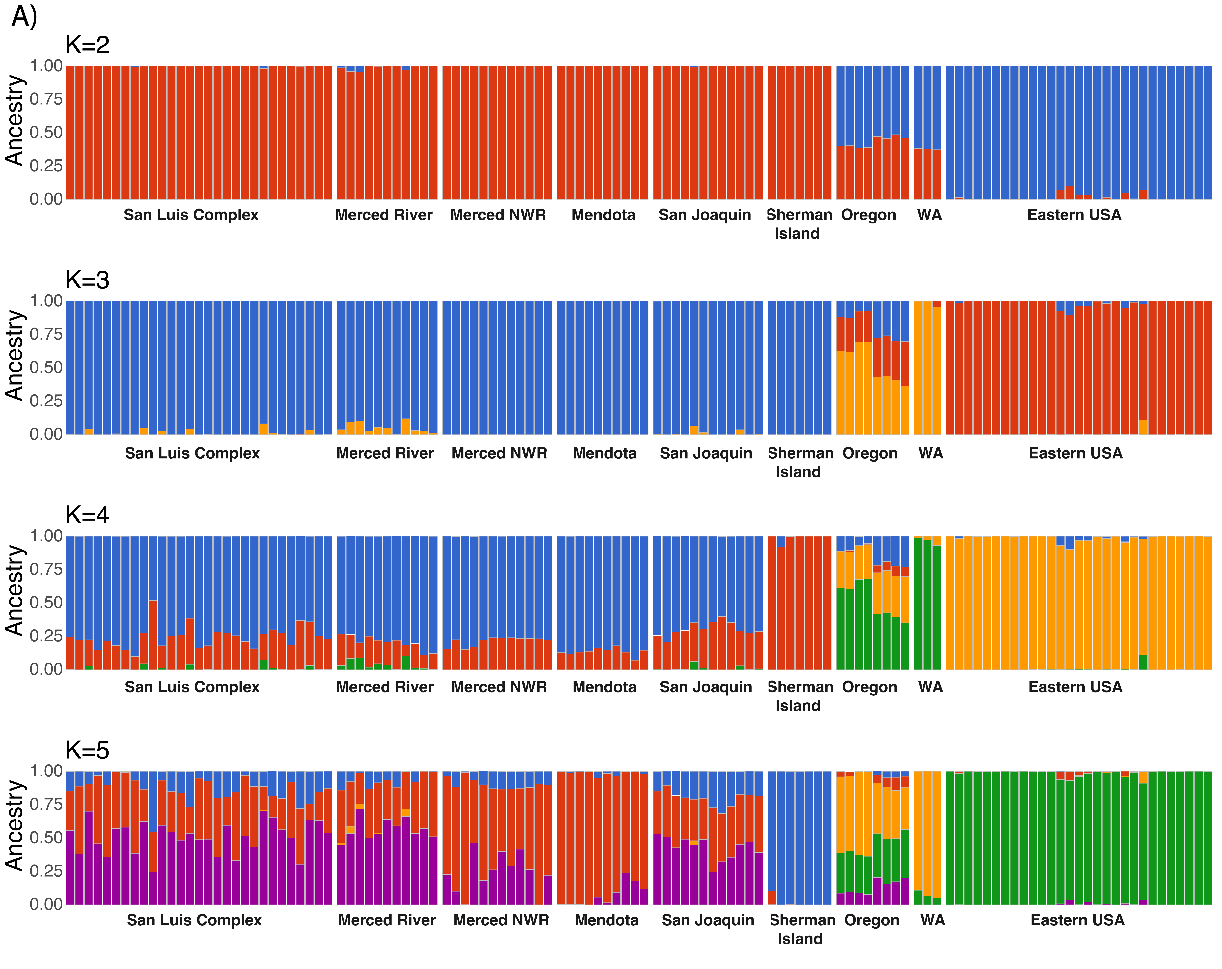

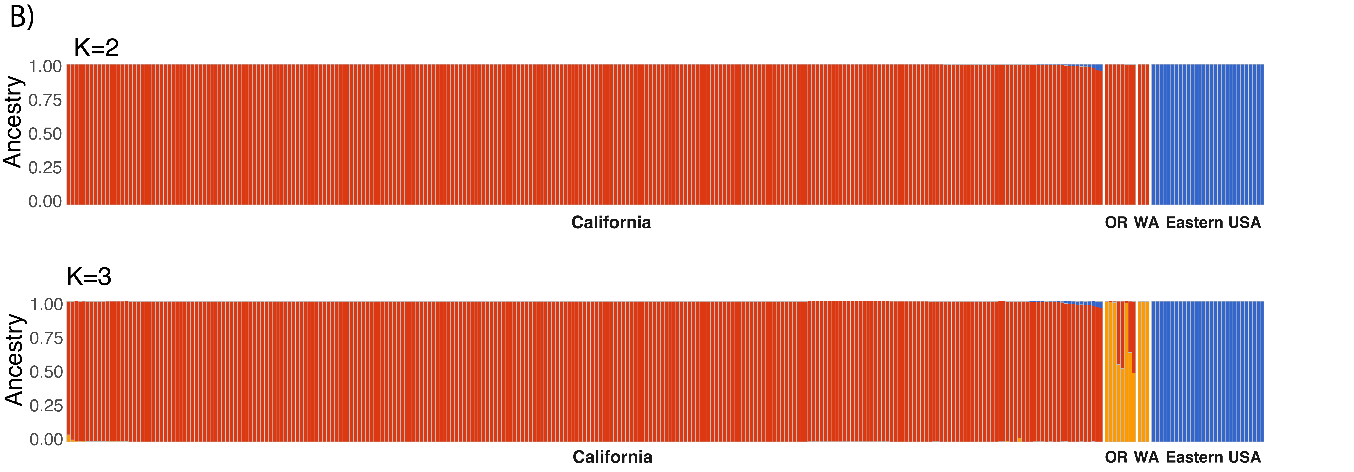


**Fig. S2** structure bar plots for **A)** the full 6,809 SNP dataset at *K* = 2 through *K* = 5 and **B)** the 403 SNP loci filtered for private alleles in East vs. West at *K* = 2 and *K* = 3. All plots were generated with 10 iterations for each *K*, a burn-in period of 250,000, and 1,000,000 MCMC.
